# Unravelling the Efficacy of Internal Quilting Sutures vs Doxycycline Instillations in Preventing Seroma Formation After Gynecomastia Surgery

**DOI:** 10.1093/asjof/ojae078

**Published:** 2024-09-10

**Authors:** Karthik Ramasamy, Abisshek Raj Alagarasan, Hitesh Gupta, Anjana Elangovan, Yash Thakkar, Kiran Silwal, Sujoy Kumar Chatterji, Sri Latha Jammu

## Abstract

**Background:**

Seroma frequently presents as a challenge, following gynecomastia correction surgery. This calls for percutaneous aspiration of accumulated fluid, from the iatrogenic dead space. The authors utilized internal quilting sutures and doxycycline instillation to analyze and compare their roles in seroma prevention.

**Objectives:**

To compare the efficacy of intraoperative internal quilting sutures and doxycycline instillation, in preventing seroma formation and recurrence after gynecomastia surgery.

**Methods:**

After local review board and ethics committee approval, the authors conducted this prospective single-center study of 120 gynecomastia patients with Rohrich's Grades I, II, and III, who underwent surgery between October 2023 and March 2024. Those belonging to Rohrich's Grade IV were excluded. Before surgery, the patients were divided into 3 cohorts of 40 individuals using a computerized randomization protocol. Cohort 1 underwent doxycycline instillation, Cohort 2 underwent internal quilting sutures, and no intervention was carried out in Cohort 3. Seroma diagnosis was confirmed clinically and the data were analyzed. All the patients were followed up for a month.

**Results:**

The incidence of seroma formation, volume of seroma fluid removed, and the number of visits for seroma care were statistically found to be the least in the quilting group compared with the doxycycline and control groups. Univariate logistic regression analysis revealed that patients belonging to both doxycycline and control groups showed significantly higher risk for seroma formation with an odds ratio of 4.705 and 6.524, respectively.

**Conclusions:**

Doxycycline instillation was less effective than internal quilting sutures in preventing seroma formation. Internal quilting sutures are a safe effective, and undemanding adjuvant technique to reduce the rate of formation and recurrence of seroma, after gynecomastia surgery.

**Level of Evidence: 2:**

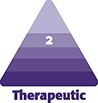

Gynecomastia is characterized by benign enlargement of breast tissue in males because of the proliferation of ductal, stromal, or fatty tissue.^1^ The prevalence of gynecomastia ranges from 40% to 60% in adult males.^2^ Current methods of surgical correction for gynecomastia include a combination of liposuction with gland removal with varying choices of incisions, preservation of nipple areola complex in varying thicknesses, and fat grafting along the pectoralis muscle border to name a few.^3^ The most common complication of gynecomastia surgery is hematoma formation followed by seroma formation, which ranges between 2% and 4% and necessitates percutaneous aspiration, often multiple times in the postoperative course.^4,5^ Seroma is defined pathologically as an abnormal accumulation of serous lymphatic fluid and plasma in the dead space. Seroma can cause both patient anxiety and discomfort thus increasing the patient's hospital visits, costs, and recovery time.^6^ An important reason for seroma to occur is the dead space formation that follows the excision of glandular and fatty tissue. Pathophysiologically, it is associated with the disruption of lymphatic channels, shearing forces between the flap and fascia, and the release of inflammatory mediators.^7,8^ Internal quilting sutures have been reported in different types of surgeries such as abdominoplasty, facelift, and thigh lift to decrease the dead space, and thus, the authors have extended this to gynecomastia surgeries to see whether this prevents seroma formation and reduces its magnitude.^9,10^ Instillation of injectable doxycycline as a sclerosing agent has been used for a long time in abdominal wall surgeries, thoracic surgery, and breast surgery for the same purpose.^11-13^ The authors after much perusal found paucity in literature comparing the supportive roles of internal quilting sutures and doxycycline instillation sclerotherapy in gynecomastia surgery.

The key objective of this study is to assess and compare the effectiveness of internal quilting sutures and doxycycline instillation sclerotherapy in the prevention of both the formation and recurrence of postoperative seromas in gynecomastia surgery.

## METHODS

Upon institutional review board and local ethical committee approval, using a computerized randomization program, power analysis was carried out to select 120 patients for this prospective cohort study. The power of this study was found to be 80%. The study period ranged between October 2023 and March 2024. The age range of the study participants was 14 to 53 years. Informed consent was obtained from patients for participation in this study and the use of their clinical photographs for research and publication purposes. The primary outcome of this study is to assess the incidence of seroma across internal quilting suture, doxycycline, and the control groups.

Secondary endpoints were postoperative outcomes, such as the total volume of seroma drained, number of visits for seroma care, the final postoperative day (POD) of seroma care, and complication rates, including hematoma, nipple necrosis, and complications of seroma such as infection/cellulitis, contour deformities, and pain.

Grading of patients was carried out using Rohrich classification,^14^ which is as follows:

- Grade I: minimal hypertrophy without ptosis
- Grade II: moderate hypertrophy without ptosis
- Grade III: severe hypertrophy with Grade I ptosis
- Grade IV: severe hypertrophy with Grade II or III ptosis

### Inclusion and Exclusion Criteria

Patients with Rohrich's gynecomastia Grades I, II, and III undergoing surgery were included in this study.^14^

Patients belonging to Rohrich's Grade IV gynecomastia, those unwilling to participate, and the candidates with unilateral gynecomastia were excluded from this study.

The authors’ decision to exclude Grade IV stems from the idea that a severe degree of ptosis, skin laxity, gland volume, and also the commonly associated higher BMI (>28) observed in Rohrich's Grade IV, most likely contribute to larger dead space cavities and a disproportionate progression of associated complications when compared with the other grades. Hence, this exclusion was carried out to avoid selection bias in the study, despite the randomization.

The authors believe that this selection criterion is necessary to minimize group variability so that statistically valid conclusions can be drawn.^15^

### Randomization and Blinding

Patients were categorized into 3 groups of 40 preoperatively into internal quilting, doxycycline sclerotherapy, and the control group. This was carried out using a computerized randomization program that helped in both random unlabeled allotment of groups and blinding patients and the other investigators as to which treatment arm each patient belonged to. All the surgeries were performed by a single surgeon at our center.

### Surgical Technique

Preoperative markings were made with the patient in standing position. The gland position and excess fat on the anterior and lateral chest walls were marked. All surgeries were performed by a single surgeon at our center under general anesthesia. Antibiotic prophylaxis was routinely given at the time of induction. The patient was placed in stable supine position with arms abducted, and a 5 mm stab incision was made at the highest point on the anterior axillary line along the axillary crease. Tumescent infiltration of 15 mL lidocaine (2%), 10 mL ropivacaine (0.5%), triamcinolone acetonide 10 mg/L, and 2 mL adrenaline (1 in 1000) in 1 L of Ringer lactate was given through the axillary incision. Triamcinolone is avoided in diabetic patients, for its hyperglycemic effect. Liposuction of excess fat from the anterior and lateral chest was performed using ultrasound-assisted liposuction (VASER) followed by power-assisted liposuction. Either a 4 or 5 mm blunt tip cannula was used for liposuction. The endpoint of liposuction was determined by the desired masculine chest shape. An inferolateral periareolar incision was made for gland excision. The anterior gland attachment from the skin is released with scissors. The gland is grasped with Allis forceps, excised, and delivered out using the pull-through technique. A similar procedure is then repeated on the other side. Bipolar cautery was used with caution to achieve hemostasis. The skin was closed in layers using 4-0 nylon (polyamide). On an average, all the surgeries were performed within 1 h and 16 min. No drains were used in any of the patients. All patients were routinely administered intravenous tranexamic acid (Video).

### Group 1: Doxycycline Instillation

This group consisted of 40 patients in whom 100 mg of doxycycline, reconstituted with 10 mL of sterile water (doxycycline 100 mg/vial lyophilized) was sprayed into the operated cavity through the periareolar incision and retained for 10 min, then wiped out through the same incision with a sterile gauze.

### Group 2: Internal Quilting Sutures

This group consisted of 40 patients in whom internal quilting sutures were used. This technique adds extra 10 min to the corrective gynecomastia surgery when incorporated as an extra step. Following liposuction and gland removal, firstly, the lower border of pectoralis major was marked, and then, a progressive 5-point internal quilting sutures were placed between the periareolar thoracic flap and the pectoralis fascia using 3.0 polyglactin sutures. These 5 points are marked as:

Point 1: skin where the pectoralis major curves toward the axilla

Point 2: the inferior border of pectoralis major muscle above the Nipple Areolar Complex (NAC)

Point 3: medial to the NAC

Point 4: inferior to the NAC

Point 5: inferolateral to the NAC

### Group 3: No Intervention

The final group included 40 patients in whom neither quilting sutures nor doxycycline instillation was used after liposuction, gland removal, and hemostasis.

### Follow-Up

All the patients were made to wear fitting compression garments immediately after surgery for 30 days, and this did not differ between groups. The patients were then followed up on PODs 3, 7, 14, and 30 for seroma collection. Postoperative seroma collection was confirmed clinically and then aspirated using an 18 G needle inserted percutaneously at the dependent part of the clinically significant seroma site, with the patient in sitting position. Lymphatic massage was also started 2 weeks postoperatively for all the patients up to 1 month.

### Clinical Definition and Examination of Seroma

Seroma is clinically defined by the presence of fluctuant serous fluid collection, creating a waterbed effect at the surgical site during the postoperative period.^16^

The authors considered an aspirated fluctuant fluid volume of 10 mL or more (≥10 mL) as clinically significant seroma.

### Statistical Analysis

The presentation of the categorical variables was carried out in the form of numbers and percentages. On the other hand, the quantitative data with normal distribution were presented as the means ± standard deviation (SD) and the data with non-normal distribution as median with 25th and 75th percentiles (interquartile range). In the cases in which the data were not normal, we used nonparametric tests. The following statistical tests were applied to the results:

1. The comparison of the quantitative variables was analyzed using analysis of variance (ANOVA; for normally distributed data) and the Kruskal–Wallis test (for non-normally distributed data). For all values that had a significant *P*-value of <.05 on ANOVA, a post hoc test (Bonferroni correction) was applied. For all not normally distributed data that had a significant *P*-value of <.05, a post hoc analysis by Dunn's multiple pairwise comparison test was carried out.
2. The comparison of the qualitative variables was analyzed using the *χ*^2^ test. If any cell had an expected value of <5, then Fisher's exact test was used.
3. Univariate logistic regression was used to identify the significant effect of interventions on seroma.
4. The data entry was carried out in a Microsoft Excel (version 2.87(24071013)) spreadsheet, and the final analysis was carried out with the use of Statistical Package for Social Sciences software (IBM manufacturer, Chicago, IL; version 25.0). For statistical significance, a *P*-value of <.05 was considered statistically significant.
5. Interrater kappa agreement was used to assess the strength of agreement between the 2 observers.

## RESULTS

### Patient and Study Characteristics

**Figure 1.**
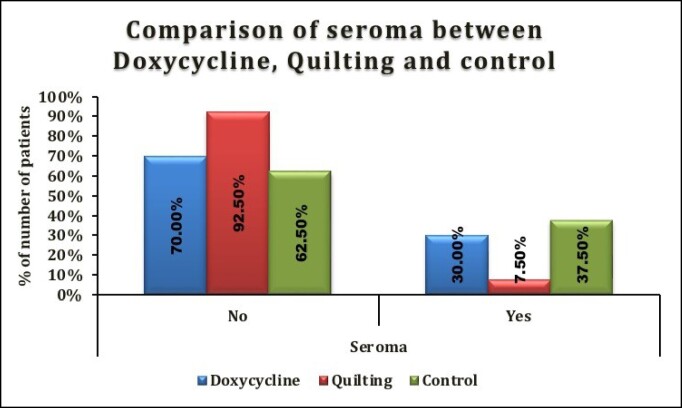
Comparison of incidence of seroma across groups.

**Figure 2.**
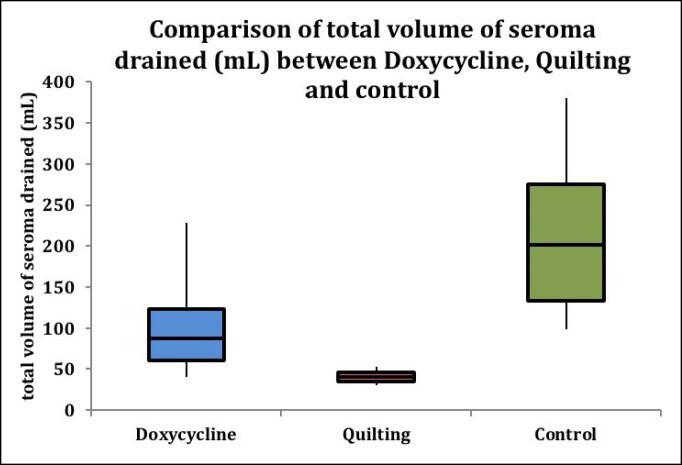
Box whisker plot graph comparing the total volumes of seroma.

Two independent observers from the same center examined the patients and recorded their findings. Clinical examination, aspiration, and data entry were carried out by 2 examiners from the same center. It is important to note these investigators were also blinded to the treatment arm being studied. There was good interobserver agreement (*κ* = 0.87). The mean age of the study population was 29.61. The mean BMI of the total study group was 26.37. Age and BMI distribution across the 3 groups are displayed in Table 1. No significant difference was observed for age (*P* = .705) and BMI (*P* = .725) between the groups (Table 1, Figure 1). The comorbidities reported in our study were diabetes mellitus and hypertension. The distribution of diabetes mellitus and hypertension was comparable across the groups. Diabetes mellitus was present in 5 (12.50%) of the doxycycline group, 7 (17.50%) of the quilting group, and 5 (12.50%) of the control group (*P* = .76). Hypertension was reported in 7 (17.50%) of the doxycycline group, 6 (15%) of the quilting group, and 4 (10%) of the control group (*P* = .619; Table 2, Figure 2). The time taken for surgery was found to be comparable across the groups (*P* = .851). The mean time taken for surgery was found to be 75.4 min. Patients were evaluated for postoperative pain by Numerical Rating Scale (NRS) scoring system (0-10).^17^

**Table 1.** Comparison of Demographic Characteristics Between Doxycycline, Quilting, and Control Groups

Demographic characteristic	Doxycycline (*n* = 40)	Quilting (*n* = 40)	Control (*n* = 40)	*P*-value
Age (years)	29.78 ± 7.48	30.22 ± 9.04	28.82 ± 6.08	.705^a^ Doxycycline vs quilting: .962 Doxycycline vs control: .843 Quilting vs control: .691
BMI (kg/m²)	26.69 ± 3.37	26.32 ± 3.62	26.1 ± 2.87	.725^a^ Doxycycline vs quilting: .876 Doxycycline vs control: .706 Quilting vs control: .95

^a^ANOVA.

**Table 2.** Comparison of Comorbidities Between Doxycycline, Quilting, and Control

Comorbidities	Doxycycline (*n* = 40)	Quilting (*n* = 40)	Control (*n* = 40)	*P*-value
Diabetes mellitus	5 (12.50%)	7 (17.50%)	5 (12.50%)	.76^a^ Doxycycline vs quilting: .531^a^ Doxycycline vs control: 1^a^ Quilting vs control: .531^a^
Hypertension	7 (17.50%)	6 (15%)	4 (10%)	.619^a^ Doxycycline vs quilting: .762^a^ Doxycycline vs control: .518^b^ Quilting vs control: .737^b^

^a^*χ*^2^ test. ^b^Fisher's exact test.

Clinical photographs of 2 patients showing before and after outcomes are provided (Supplemental Figures 1, 2).

### Grade of Gynecomastia

In the doxycycline group, 6 (15%) presented with Grade 1, 17 (42.50%) with Grade 2, and 17 (42.50%) with Grade 3. In the quilting group, 2 (5%) presented with Grade 1, 24 (60%) with Grade 2, and 14 (35%) with Grade 3. In the control group, 3 (7.50%) had Grade 1, 25 (62.50%) had Grade 2, and 12 (30%) had Grade 3. No significant difference was seen in grading between the doxycycline, quilting, and control groups (*P* = .309).

### Complications

Hematoma formation was seen in 3 patients in total, 1 (2.5%) from the doxycycline group and 2 (5%) from the control group. In total 4 patients developed nipple necrosis, 3 (7.5%) in the doxycycline group and 1 (2.5%) in the control group.

These results indicate that the occurrence of hematoma (*P* = .772) and nipple necrosis (*P* = .322) were comparable across the groups. Infection/cellulitis was seen in 2 patients, 1 in the doxycycline group (2.5%) and 1 in the control group (2.5%), both of which were resolved by POD 7 (*P* = 1). None of the patients developed contour irregularities, panniculitis, or fat necrosis. The NRS pain score mean expressed in integers (0-10) was (mild) NRS-3 across the 3 study groups. The score for pain was comparable across groups, and its mean was found to be (mean NRS pain score) NRS-3 across the groups (*P* = .645).

### Incidence of Seroma

A total of 30 out of 120 patients developed seroma in this study (25%). A significant difference was seen in the incidence of seroma between the doxycycline, internal quilting, and control groups (*P* = .006). Seroma was present in 12 (30%) patients of the doxycycline group, 3 (7.50%) patients from the internal quilting group, and 15 (37.50%) from the control group. The internal quilting group had a significantly lower incidence of seroma compared with the doxycycline and control groups (*P* = .006; Figure 1, Table 3).

**Table 3.** Comparison of Seroma Between Doxycycline, Quilting, and Control

Seroma	Doxycycline (*n* = 40)	Quilting (*n* = 40)	Control (*n* = 40)	*P*-value
No	28 (70%)	37 (92.50%)	25 (62.50%)	.006^a^ Doxycycline vs quilting: .02^b^ Doxycycline vs control: .478^a^ Quilting vs control: .003^b^
Yes	12 (30%)	3 (7.50%)	15 (37.50%)	
Total	40 (100%)	40 (100%)	40 (100%)	

^a^*χ*^2^ test. ^b^Fisher’s exact test.

### Total Volume of Seroma Across Groups

A significant difference was seen in the total volume of seroma drained between the doxycycline, quilting, and control groups (*P* = .0006). The median (25th-75th percentiles) volume of seroma drained was 87.5 mL (61-122.75 mL) for the doxycycline group, 40 mL (35-46.5 mL) for the quilting group, and 201 mL (133-275.5 mL) for the control group. The control and doxycycline group had a significantly higher volume of seroma drained compared with the quilting group (Figure 2, Table 4).

**Table 4.** Comparison of Total Volume of Seroma Drained (mL) Between Doxycycline, Quilting, and Control Groups

Total volume of seroma drained (mL)	Doxycycline (*n* = 12)	Quilting (*n* = 3)	Control (*n* = 15)	*P*-value
Median (25th-75th percentile)	87.5 (61-122.75)	40 (35-46.5)	201 (133-275.5)	.0006^a^ Doxycycline vs quilting: .124 Doxycycline vs control: .005 Quilting vs control: .001

^a^Kruskal–Wallis.

### Total Follow-Up Visits for Seroma Care

**Figure 3.**
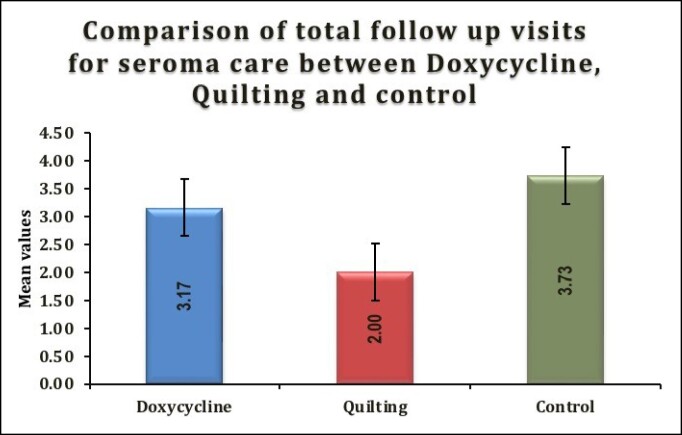
Comparison of total follow-up visits for seroma care between doxycycline, quilting, and control.

The mean ± SD number of follow-up visits in the doxycycline group was 3.17 ± 0.94, in the quilting group, it was 2 ± 1, and in the control group, it was 3.73 ± 0.7, with a significant overall *P*-value of .008. When comparing the groups pairwise, the *P*-value for doxycycline vs quilting was .093, indicating no significant difference between these 2 groups. The *P*-value for doxycycline vs control was .201, also indicating no significant difference. However, the comparison between quilting vs control showed a significant difference with a *P*-value of .007 (Table 5, Figure 3).

**Table 5.** Comparison of Total Follow-up Visits for Seroma Care Between Doxycycline, Quilting, and Control

Variable	Doxycycline (*n* = 12)	Quilting (*n* = 3)	Control (*n* = 15)	*P*-value
Total follow-up visits for seroma care	3.17 ± 0.94	2 ± 1	3.73 ± 0.7	.008^a^ Doxycycline vs quilting: .093 Doxycycline vs control: .201 Quilting vs control: .007

^a^ANOVA.

### Final Postoperative Day of Seroma Care

The median (25th-75th percentile) final POD of seroma drainage was 30 days (26-30 days) for the doxycycline group, 14 days (10.5-22 days) for the quilting group, and 30 days (14-30 days) for the control group. These results indicated that the duration of seroma drainage was similar across the groups (Table 6).

**Table 6.** Comparison of Final POD of Seroma Drainage Between Doxycycline, Quilting, and Control

Final POD of seroma drainage	Doxycycline (*n* = 12)	Quilting (*n* = 3)	Control (*n* = 15)	*P*-value
Median (25th-75th percentile)	30 (26-30)	14 (10.5-22)	30 (14-30)	.25^a^ Doxycycline vs quilting: .097 Doxycycline vs control: .656 Quilting vs control: .155

POD, postoperative day. ^a^Kruskal–Wallis test.

### Univariate Logistic Regression Analysis

To recognize the relationship between seroma formation and the intervention type, a univariate regression analysis was carried out. The doxycycline and control groups were significant factors affecting seroma formation. Patients belonging to both doxycycline and control groups showed a significantly high risk for seroma formation with an odds ratio of 4.705 and 6.524, respectively (Table 7).

**Table 7.** Univariate Logistic Regression to Identify the Significant Effect of Interventions on Seroma

Variable	Beta-coefficient	Standard error	*P*-value	Odds ratio	Odds ratio lower bound (95%)	Odds ratio upper bound (95%)
Age (years)	−0.013	0.028	0.642	0.987	0.934	1.043
BMI (kg/m²)	0.012	0.064	0.848	1.012	0.892	1.149
Grading						
Grade I				1.000		
Grade II	−0.350	0.817	0.668	0.705	0.142	3.492
Grade III	1.010	0.805	0.209	2.746	0.567	13.290
DM	−0.026	0.604	0.966	0.974	0.298	3.183
HTN	0.607	0.556	0.274	1.836	0.618	5.455
Nipple necrosis	0.000	1.174	1.000	1.000	0.100	9.993
Infection/cellulitis	−1.182	2.391	0.621	0.307	0.003	33.271
Hematoma formation	0.417	1.243	0.737	1.517	0.133	17.354
Group						
Quilting				1.000		
Doxycycline	1.549	0.662	0.019	4.705	1.285	17.222
Control	1.876	0.653	0.004	6.524	1.814	23.473

## DISCUSSION

The surgical correction of gynecomastia has been refined over the years and now aims at minimal manipulation and an aesthetically appealing male chest juxtaposed with an optimal recovery time. Seroma formation after gynecomastia surgery is a commonly encountered menace that is known to hinder the process of recovery and thereby the outcome of aesthetic results. It can be a real spot of bother for both the patients and surgeons. In this study, only 3 patients developed hematomas, most likely owing to the use of adrenaline in tumescent liposuction, and avoidance of superficial liposuction.^18,19^ Bipolar cautery was sparingly used with care to achieve hemostasis as studies have reported the extensive use of electrocautery in dissection could increase the incidence of seroma.^20^ No patient developed contour irregularities. On average, all surgeries were performed within 1 h and 16 min. The patient satisfaction rates were high in our study where 75% of patients did not develop seroma during the postoperative period. Overall, 25% of patients developed seroma in this study. Seroma was found in 12 (30%) patients of the doxycycline group, 3 (7.50%) patients from the internal quilting group, and 15 (37.50%) from the control group. The internal quilting group had a significantly lower incidence of seroma compared with the others (*P* = .006).

Moreover, the control and doxycycline groups had significantly higher volumes of seroma drained compared with the internal quilting group (*P* = .0006). The comparison between postoperative follow-up visits for seroma care between the quilting and control groups showed a significant difference (*P* = .007). There were fewer follow-up visits for seroma care specifically in the quilting group. The authors have utilized a novel 5-point internal quilting sutures technique to minimize the dead space. This is achieved through multiple anchor points. Further, they hypothesize that these sutures maintain a robust connection between the skin flap and the pectoralis fascia. This prevents movement and thereby decreases the friction of 1 tissue plane over the other. In addition, this allows for better compartmentalization of the fluid collected underneath the flap thus facilitating better absorption and quicker recovery from third spacing. A similar theory was proposed in a study by Andrades et al in abdominoplasty where progressive tension sutures were utilized for the same reason.^7^ Internal quilting also promotes better redraping of redundant skin to the pectoralis fascia. Murugesan and Karidis in their study have made use of external quilting sutures to prevent hematoma and seroma formation.^21^ The authors state that the disadvantage of external quilting lies in the fact that it may leave behind external marks and postinflammatory hyperpigmentation, especially in skin of color. This leads to a less aesthetically appealing appearance of the chest. The external sutures have to be removed in the postoperative period, which always carries a risk of button-hole-like scarring at the site where the external suture bites were taken. The internal quilting sutures have the advantage of no external quilting scars or postinflammatory hyperpigmentation, thereby reducing the need for any scar revision procedures at these sites. Although this technique adds extra 10 min to the steps of gynecomastia surgery, it helps alleviate the recovery time, and cost incurred for hospital visits owing to seroma aspiration care and ameliorates the aesthetic chest appearance after surgery, thereby resulting in an overall improved end result.

In this study, doxycycline instillation sclerotherapy was applied with the rationale that doxycycline, apart from its antibiotic effect has been demonstrated to increase growth factor like activity in cells which also leads to local fibrin deposition and fibroblast proliferation that approximates the dead space, thus preventing seroma formation.^22^ Seroma incidence was seen in 12 patients from the doxycycline group (30%), with the median total aspirated seroma volume for this group being 87.5 mL when compared with the control group (201 mL) albeit higher than the internal quilting group. These findings for the doxycycline group are not statistically or clinically relevant with the purview of preventing seroma formation and its associated complications. Similar findings were reported in a study by Turk et al in hernia surgery with no significant differences regarding seroma prevention with topical tetracycline.^23^ Rice et al found more seroma formation 2 weeks postoperatively with the intraoperative tetracycline sclerotherapy following mastectomy.^24^ However, doxycycline has been reported to be useful in the management of seromas in small-scale studies with strong success, minimal complications, and cost-to-benefit comparisons.^12,13^ In our study, univariate logistic regression analysis showed high risk and odds ratios corresponding to increased risk for seroma formation in both doxycycline and control groups. Thus, the 5-point internal quilting technique is a safe, reproducible, effective, and useful adjuvant step that could be added to prevent and extenuate seroma formation without the risk of unnecessary external scarring in the management of gynecomastia.

### Strengths and Limitations

The authors deem the efforts taken with the levels of blinding, randomization, and a good interobserver agreement as strengths of this study. The limitations of this study include a small number of patients, apart from being a single-center study where all patients were operated by a single surgeon. The contact time and frequency for doxycycline instillation to act as a sclerosant in reducing seroma are also not well studied. Further studies with high levels of evidence are required to compare conclusively the efficacy of doxycycline sclerotherapy vs no interventions across all grades of gynecomastia.

## CONCLUSIONS

It was found that patients belonging to both doxycycline and control groups showed a significantly higher risk for seroma compared with the quilting group. In this study, doxycycline instillation was found to be less effective than quilting sutures in preventing seroma formation. Further, the quilting sutures group outdid the other 2 groups in all the significant and clinically relevant parameters used in this study. The authors conclude that the quilting sutures technique is a very safe, quick, inexpensive, effective, and easy adjuvant technique in preventing and reducing postoperative seroma formation in corrective gynecomastia surgery.

## Supplemental Material

This article contains supplemental material located online at www.asjopenforum.com.

## Supplementary Material

ojae078_Supplementary_Data
