# Supplementary figures and images for: Unravelling the Efficacy of Internal Quilting Sutures vs Doxycycline Instillations in Preventing Seroma Formation After Gynecomastia Surgery
Source: Aesthet Surg J Open Forum. 2024 Sep 10;7:ojae078. doi: 10.1093/asjof/ojae078 (PMC11852251; doi:10.1093/asjof/ojae078)

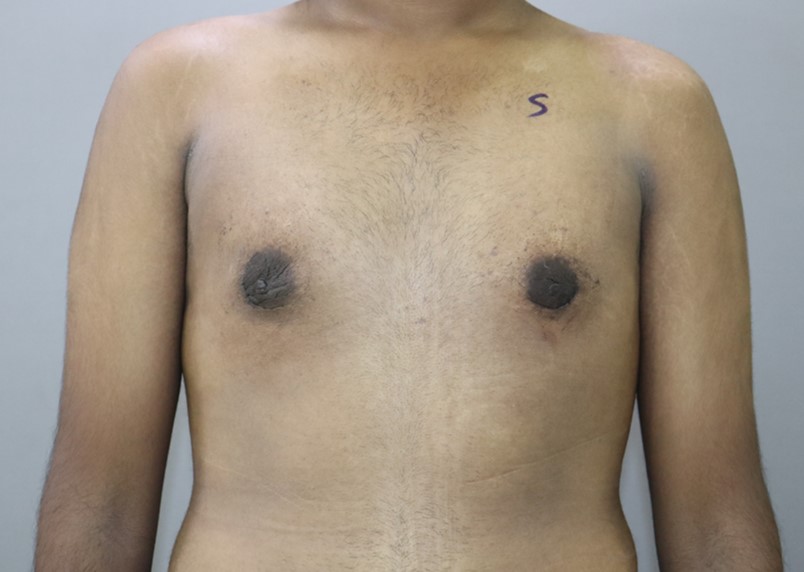

Supplement: ojae078_Supplementary_Data [file ojae078_Supplementary_Data.zip › 2A AFTER.jpg]

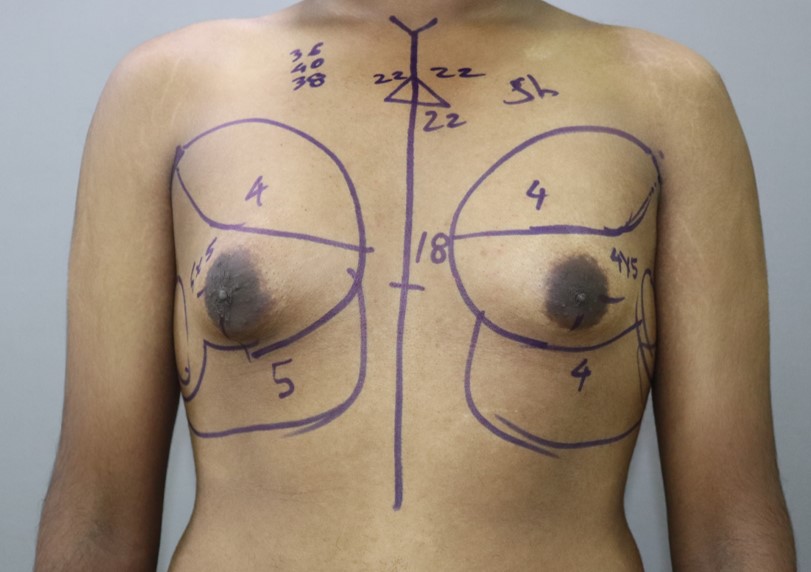

Supplement: ojae078_Supplementary_Data [file ojae078_Supplementary_Data.zip › 2A BEFORE.jpg]

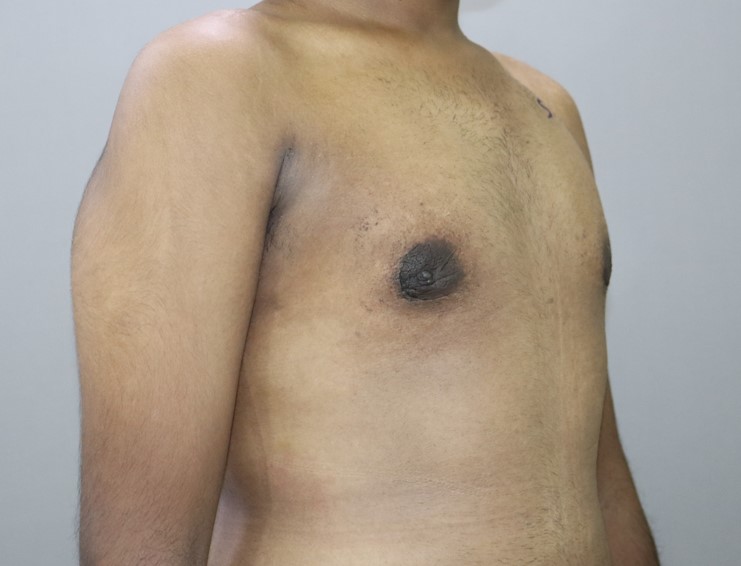

Supplement: ojae078_Supplementary_Data [file ojae078_Supplementary_Data.zip › 2B AFTER.jpg]

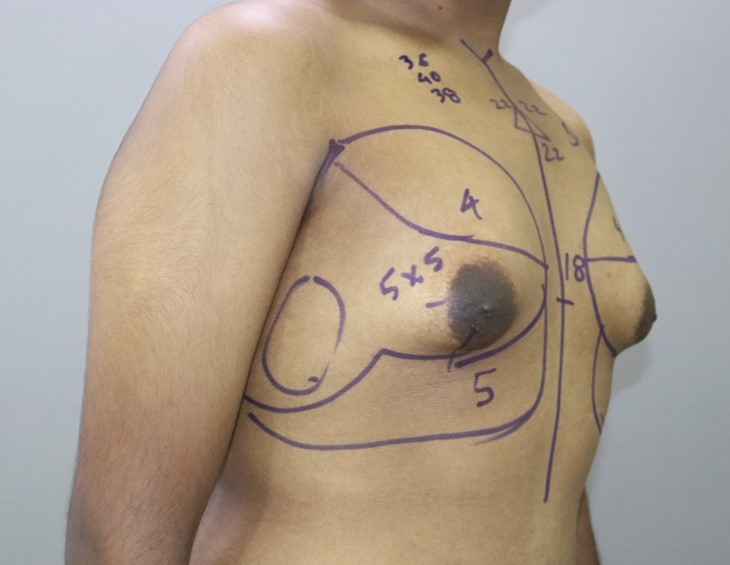

Supplement: ojae078_Supplementary_Data [file ojae078_Supplementary_Data.zip › 2B BEFORE.jpg]

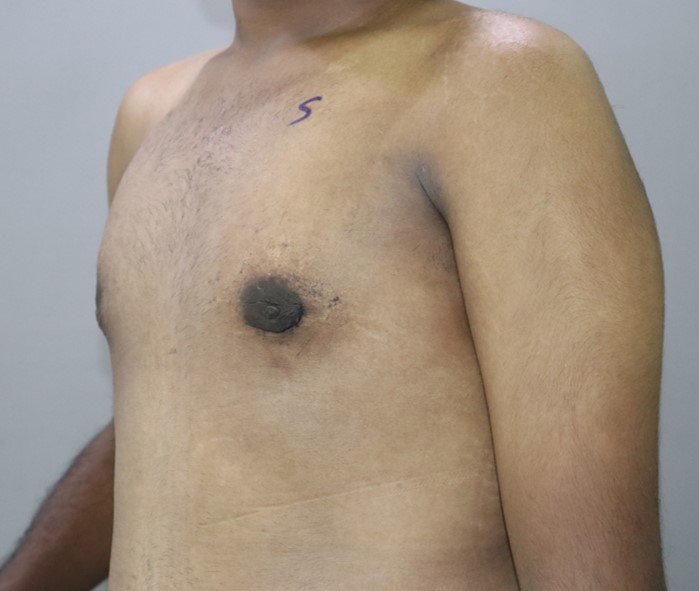

Supplement: ojae078_Supplementary_Data [file ojae078_Supplementary_Data.zip › 2C AFTER.jpg]

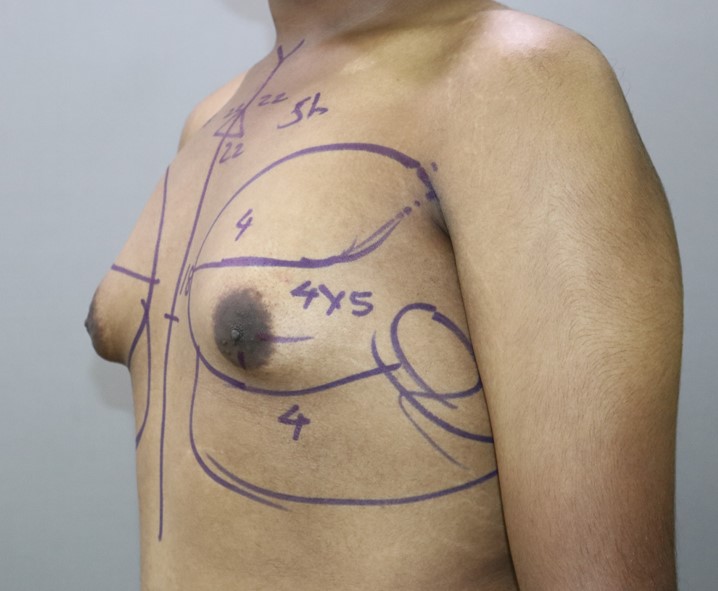

Supplement: ojae078_Supplementary_Data [file ojae078_Supplementary_Data.zip › 2C BEFORE.jpg]

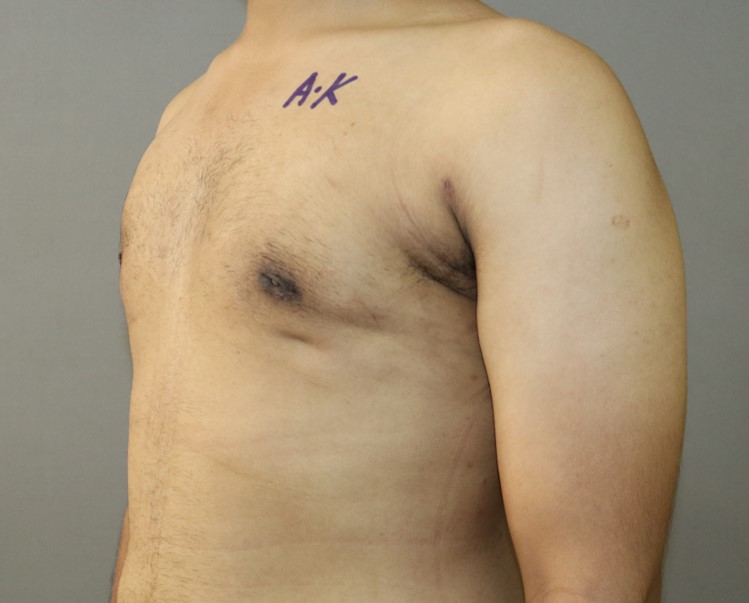

Supplement: ojae078_Supplementary_Data [file ojae078_Supplementary_Data.zip › 1 B AFTER.jpg]

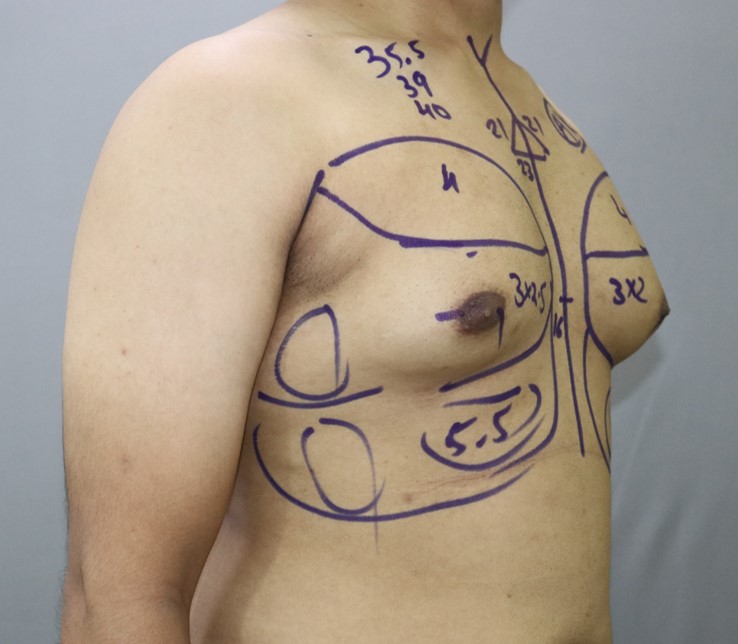

Supplement: ojae078_Supplementary_Data [file ojae078_Supplementary_Data.zip › 1 C BEFORE.jpg]

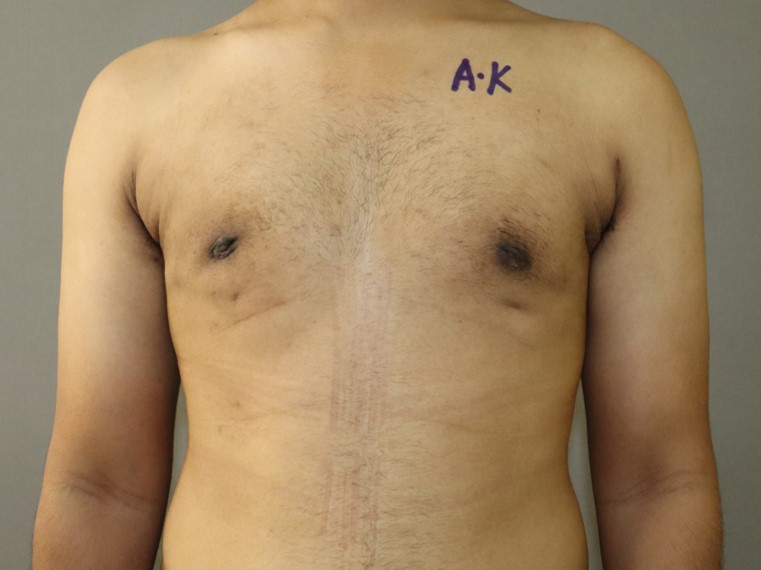

Supplement: ojae078_Supplementary_Data [file ojae078_Supplementary_Data.zip › 1A AFTER.jpg]

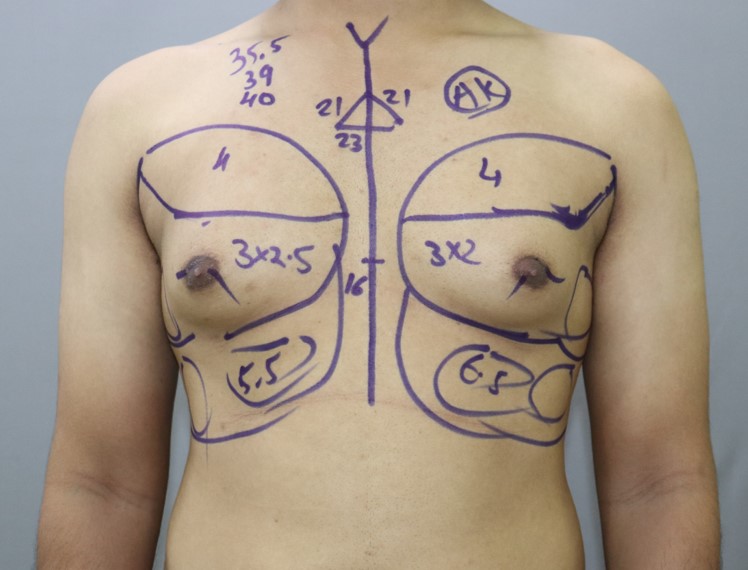

Supplement: ojae078_Supplementary_Data [file ojae078_Supplementary_Data.zip › 1A BEFORE.jpg]

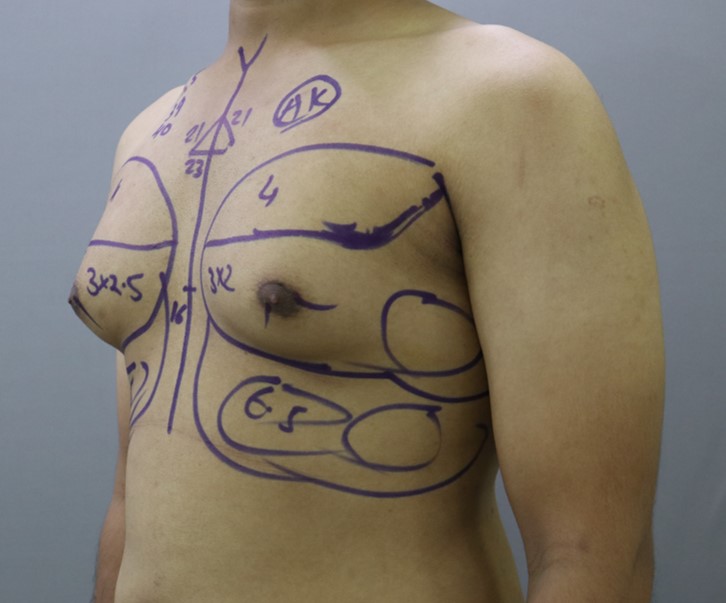

Supplement: ojae078_Supplementary_Data [file ojae078_Supplementary_Data.zip › 1B BEFORE.jpg]

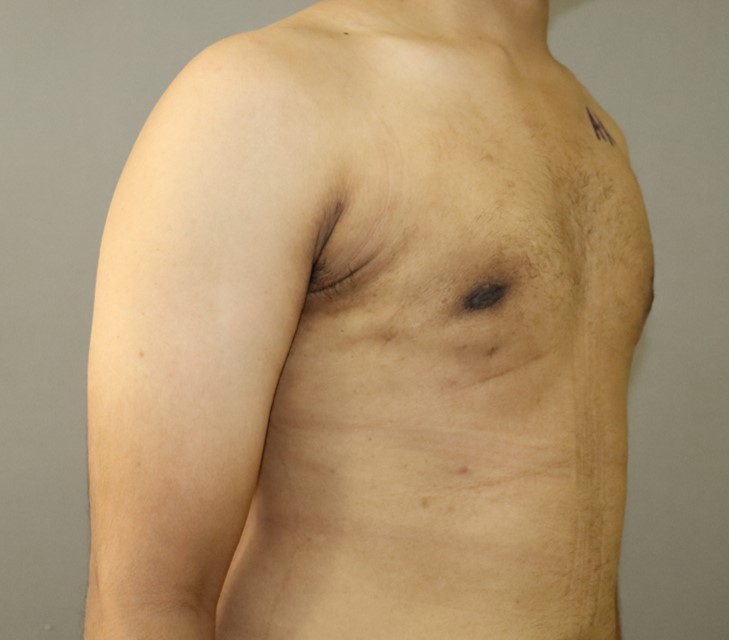

Supplement: ojae078_Supplementary_Data [file ojae078_Supplementary_Data.zip › 1C AFTER.jpg]
